
JOURNAL INFORMATION
==============================
NLM Title Abbreviation: Wirtschaftsdienst
Journal ID: Wirtschaftsdienst
NLM Journal ID: 101086612

Wirtschaftsdienst (Hamburg, Germany : 1949)

ISSN: 0043-6275

ARTICLE INFORMATION
==============================
PMCID: PMC8598270
PMID: 34812210
DOI: 10.1007/s10273-021-3045-8
Article ID: 3045
Article version: 1
Subjects: Ökonomische Trends

Unternehmen setzen verstärkt auf Crowdworking

Companies increasingly rely on crowdworking

Erdsiek Daniel Daniel.Erdsiek@zew.de

grid.13414.33 0000 0004 0492 4665 ZEW - Leibniz-Zentrum für Europäische Wirtschaftsforschung, L 7, 1, 68161 Mannheim, Deutschland
Electronic publication date: 2021 Nov 18
Print publication date: 2021
Volume: 101
Issue: 11
First page: 912
Last page: 914
Copyright: © Der/die Autor:in 2021
License: Open Access: Dieser Artikel wird unter der Creative Commons Namensnennung 4.0 International Lizenz veröffentlicht (https://creativecommons.org/licenses/by/4.0/deed.de).
Open Access wird durch die ZBW — Leibniz-Informationszentrum Wirtschaft gefördert.
License URL: https://creativecommons.org/licenses/by/4.0/

issue-copyright-statement: © The Author(s) 2021

==============================

Durch den Fortschritt digitaler Technologien sind in den vergangenen Jahren neue Formen der Erwerbsarbeit entstanden. Neben dem klassischen Arbeitsmarkt bieten nun auch Online-Plattformen die Möglichkeit zur Vermittlung von Arbeitsaufträgen. Dabei können grundsätzlich zwei Formen der plattformbasierten Erwerbsarbeit unterschieden werden: Zum einen können Online-Plattformen zur Vergabe von Arbeitsaufträgen eingesetzt werden, die physisch an einem vereinbarten Ort ausgeführt werden müssen. Einsatzbereiche dieser ortsgebundenen Plattformarbeit umfassen etwa Personenbeförderung, Logistikdienste oder Haushaltsdienste. Zum zweiten eignen sich Online-Plattformen auch für die Vergabe von Aufträgen, die über die Plattform rein internetbasiert und damit ortsunabhängig erbracht werden können. Diese Beschäftigungsform wird auch als „Crowdworking" bezeichnet und ermöglicht es Unternehmen, traditionell unternehmensinterne Aufgaben und Projekte über eine Plattform an externe Arbeitskräfte zu vergeben. Die gegen Bezahlung ausgeführten Tätigkeiten reichen dabei von einfachen Kleinstaufgaben, die nur wenige Klicks erfordern, bis hin zur Erbringung komplexer Tätigkeiten und vollständiger Projekte, z. B. im Bereich Webprogrammierung oder Design.

Auf nationaler wie auch EU-Ebene steht die Plattformarbeit verstärkt im Fokus der politischen Debatte. Im Kern geht es dabei häufig um die Frage, welcher Gestaltungsbedarf hier entstehen kann, da Plattformarbeitende unter anderem erheblichen Unsicherheiten bezüglich ihres arbeitsrechtlichen Status und ihrer sozialen Absicherung ausgesetzt sind (Bundesregierung, 2021; Berg et al., 2018). Gleichzeitig wird aber auch hervorgehoben, dass Plattformarbeitende vom niedrigschwelligen Angebot örtlich und zeitlich flexibler Erwerbsarbeit profitieren können.

Amtliche Statistiken können bislang nicht als Grundlage für die Einschätzung der gesamtwirtschaftlichen Bedeutung des Phänomens herangezogen werden. Empirische Erkenntnisse basieren daher insbesondere auf Bevölkerungsbefragungen und von Plattformbetreibenden veröffentlichten Nutzungszahlen, die allerdings oftmals auch inaktive Nutzer:innen enthalten. Repräsentative Ergebnisse zur Verbreitung der Plattformarbeit in Deutschland liefert beispielsweise die Omnibusbefragung von 10.000 Personen durch Bonin und Rinne (2017). Zur Jahresmitte 2017 führte den Ergebnissen zufolge weniger als 1 % der erwachsenen, deutschsprachigen Bevölkerung Arbeitsaufträge aus, die über das Internet oder eine App akquiriert wurden. Im Detail erbrachten 0,6 % eine ortsgebundene Plattformarbeit und 0,3 % eine ortsunabhängige Plattformarbeit (Crowdworking). Weitere Studien weisen teils deutlich höhere Nutzungsraten in Deutschland und anderen EU-Ländern aus (Pesole et al., 2018). Anhand der bislang vorliegenden Zahlen stellt die plattformbasierte Erwerbsarbeit allerdings kein Massenphänomen dar und wird überwiegend zum Nebenerwerb genutzt. Gleichwohl wird der Plattformarbeit eine hohe Wachstumsdynamik zugesprochen (Kässi et al., 2021).

Das Phänomen der Plattformarbeit wird in Deutschland vorrangig aus der Angestelltenperspektive untersucht. Hinsichtlich der Nachfrageseite der Plattformarbeit liegen daher kaum repräsentative Ergebnisse vor (Maier et al., 2017). Eine Ausnahme besteht in den regelmäßig durchgeführten Unternehmensbefragungen des Forschungsbereichs Digitale Ökonomie am ZEW Mannheim (Ohnemus et al., 2016; Erdsiek et al., 2019; Belletti et al., 2021).[1] Um die ökonomische Relevanz der ortsunabhängigen Plattformarbeit von der Unternehmensseite her abzubilden, wurden erstmals 2014 Unternehmen in der deutschen Informationswirtschaft zu ihrer Nutzung von Crowdworking-Plattformen befragt.[2] Beginnend mit 2016 wurde die Analyse um Unternehmen aus dem Verarbeitenden Gewerbe erweitert. Durch die erneute Befragung der Unternehmen in einem Abstand von zwei Jahren, ergeben sich Erkenntnisse über die Dynamik der gewerbli-

1 Um die Repräsentativität der Ergebnisse zu gewährleisten, werden die Antworten der Umfrageteilnehmenden auf die Zahl aller Unternehmen der betrachteten Branchen hochgerechnet. Im Durchschnitt beteiligten sich über 1.000 Unternehmen an einer Befragungswelle.

2 Die Informationswirtschaft umfasst die Teilbranchen: 1. IKT-Hardware, 2. IKT-Dienstleister, 3. Mediendienstleister, 4. Rechts- und Steuerberatung, Wirtschaftsprüfung, 5. Public-Relations- und Unternehmensberatung, 6. Architektur- und Ingenieurbüros, technische, physikalische und chemische Untersuchung, 7. Forschung und Entwicklung, 8. Werbung und Marktforschung, 9. sonstige freiberufliche, wissenschaftliche und technische Tätigkeiten.

Abbildung 1

**Aktuelle und zukünftige Nutzung von Crowdworking-Plattformen**

in %

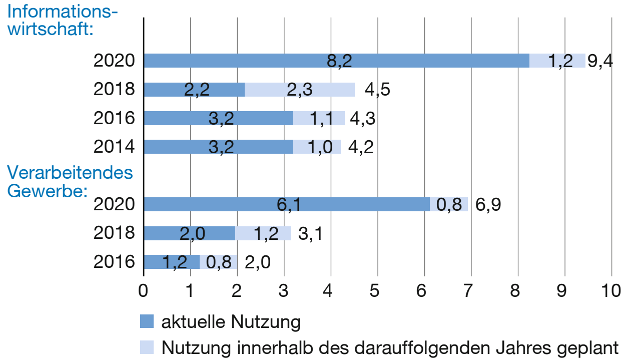

Lesehilfe: Im September 2020 gaben 8,2 % der Unternehmen in der Informationswirtschaft an, dass sie Crowdworking einsetzen. Zusätzlich planen 1,2 % der Unternehmen einen Einsatz bis Ende 2021.

Quelle: ZEW Konjunkturumfrage Informationswirtschaft.

Abbildung 2

**Mögliche Einsatzzwecke und Ziele für den Einsatz von Crowdworking**

in %

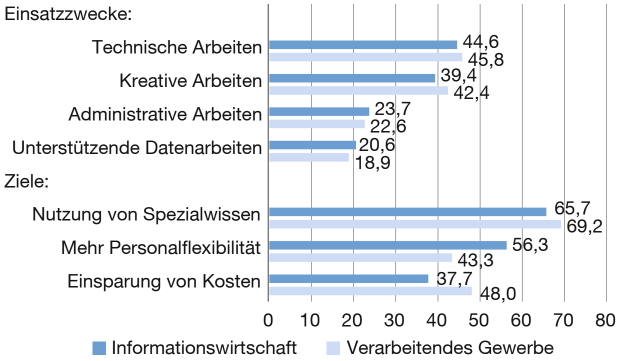

Lesehilfe: In der Informationswirtschaft sehen 44,6 % der Unternehmen, denen Crowdworking bekannt ist, technische Arbeiten als einen möglichen Einsatzzweck für Crowdworking in ihrem Unternehmen an.

Quelle: ZEW Konjunkturumfrage Informationswirtschaft.

chen Nutzung von Crowdworking in Deutschland über einen Zeitraum von mittlerweile sieben bzw. fünf Jahren.

Die Nutzung von Crowdworking-Plattformen durch Unternehmen in Deutschland ist in den vergangenen Jahren kontinuierlich gestiegen (vgl. Abbildung 1). 2020 setzten 8,2 % der Unternehmen in der Informationswirtschaft Crowdworking ein. Darüber hinaus planten 1,2 %, bis Ende 2021 mit dem Einsatz zu beginnen. In den vorherigen Befragungswellen hatten deutlich weniger Unternehmen Crowdworking eingesetzt oder den Einsatz geplant. Von 2014 bis 2018 lagen diese Anteile jeweils noch recht konstant auf einem Niveau von 4,2 % bis 4,5 %. Die (geplante) Nutzungsrate von Crowdworking-Plattformen hat sich demnach in der Informationswirtschaft zwischen 2018 und 2020 mehr als verdoppelt. Im Verarbeitenden Gewerbe setzten derweil 6,1 % der Unternehmen 2020 Crowdworking ein und 0,8 % planten den Einsatz bis Ende 2021. Ausgehend von einem Anteil von 2,0 % im Jahr 2016 ist also auch hier die (geplante) Nutzungsrate der Unternehmen in jüngster Zeit deutlich gestiegen und hat sich seit 2018 verdoppelt. Von 2018 bis 2020 ist damit in beiden Sektoren ein deutlicher Schub beim Einsatz von Crowdworking zu verzeichnen. Da die aktuellste Befragung im September 2020 durchgeführt wurde, könnte ein Teil dieses Nutzungsanstiegs auch auf coronabedingte Anpassungen der Arbeitsorganisation und einen gestiegenen Bedarf an z. B. externen IT-Spezialist:innen zurückzuführen sein.[3]

Im Fragebogen der ZEW-Erhebung erhielten teilnehmende Unternehmen eine kurze Definition des Begriffs Crowdworking, bevor sie nach der eigenen Nutzung gefragt wurden. Bei der entsprechenden Frage konnten Unternehmen dabei auch antworten, dass ihnen der Begriff bislang nicht bekannt war. Dies trifft auf rund ein Viertel in der Informationswirtschaft und ein Drittel im Verarbeitenden Gewerbe zu. Über die Bekanntheit und Nutzung von Crowdworking-Plattformen hinaus, lässt die Befragung auch Rückschlüsse über mögliche Einsatzzwecke zu. Diese sind aus Sicht der Unternehmen, denen das Phänomen Crowdworking bekannt ist, durchaus vielfältig (vgl. Abbildung 2). So zählen in der Informationswirtschaft und im Verarbeitenden Gewerbe rund 45 % der Unternehmen technische Arbeiten, z. B. in Bezug auf Internet, Programmierung und Datenanalyse, zu den möglichen Einsatzzwecken in ihrem Unternehmen. Fast ebenso viele Unternehmen sehen Potenzial für die Vergabe von kreativen Arbeiten über Crowdworking-Plattformen. Dazu zählen etwa Tätigkeiten in den Bereichen Grafikdesign, Marketing oder das Verfassen von Texten. Mit deutlichem Abstand folgen administrative Arbeiten, wie Buchführung, Kundendienst oder Projektmanagement, die für fast ein Viertel der Unternehmen ein mögliches Anwendungsgebiet darstellen. Rund jedes fünfte Unternehmen könnte sich derweil vorstellen, unterstützende Datenarbeiten auszulagern, wie etwa Kategorisierung und Tagging von Daten.

Neben den potenziellen Anwendungsgebieten variieren auch die Ziele, die Unternehmen mit einem möglichen Einsatz von Crowdworking verfolgen würden. Mit Anteilen von fast 70 % nennen die Unternehmen, denen Crowdworking ein Begriff ist, besonders häufig das Nutzen von Spezialwissen als potenzielles Hauptziel. Dem

3 In diesem Zusammenhang zeigt eine Studie auf Basis des Online Labour Index, dass auf den führenden US-Plattformen Angebot und Nachfrage im Bereich Softwareentwicklung und Technologie kurz nach dem Beginn der Corona-Pandemie schneller anstiegen als in anderen Tätigkeitsbereichen (Stephany et al., 2020).

Abbildung 3
**Hemmnisse beim Einsatz von Crowdworking**
in %

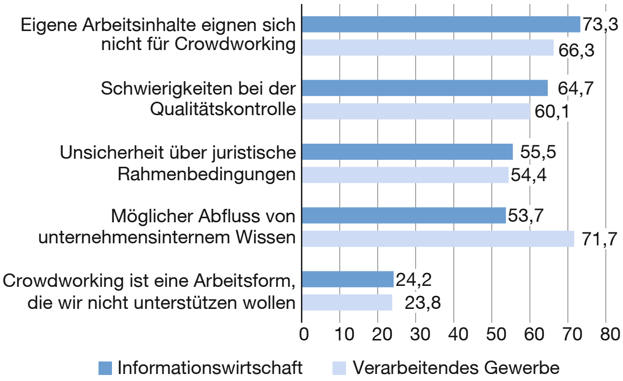

Lesehilfe: 73,3 % der Unternehmen in der Informationswirtschaft, denen Crowdworking bekannt ist, geben an, dass sich nach ihrer Einschätzung die eigenen Arbeitsinhalte nicht für Crowdworking eignen.

Quelle: ZEW Konjunkturumfrage Informationswirtschaft.

Einsatzbereich entsprechend könnte sich dieses Spezialwissen beispielsweise auf Programmierfähigkeiten oder Designkenntnisse beziehen, die nicht oder nicht in ausreichendem Maße im eigenen Unternehmen verfügbar sind. In der Informationswirtschaft würden etwa 56 % der Unternehmen durch den Einsatz von Crowdworking mehr Personalflexibilität erreichen wollen, wie etwa zum Abbau von Lastspitzen oder einer schnelleren Bearbeitung von Projekten. Erst danach folgt mit einem Anteil von knapp 38 % das Ziel, mit der Auslagerung von Arbeitsaufträgen Kosten einsparen zu können. Solche Einsparungen könnten sich etwa ergeben, weil keine Sozialabgaben und geringere Fixkosten anfallen. Für Unternehmen im Verarbeitenden Gewerbe, denen Crowdworking bekannt ist, wäre hingegen etwas häufiger die Einsparung von Kosten (48 %) ein Ziel der möglichen Crowdworking-Nutzung als die gesteigerte Personalflexibilität (43 %).

Hinsichtlich der zu erwartenden Wachstumsdynamik der plattformbasierten Erwerbsarbeit stellt sich die Frage, welche Faktoren aus der Unternehmensperspektive gegen den Einsatz von Crowdworking-Plattformen sprechen oder deren Nutzung erschweren. Aus Sicht der Unternehmen besteht das am weitesten verbreitete Hemmnis darin, dass sich die eigenen Arbeitsinhalte nicht für die Bearbeitung über eine Crowdworking-Plattform eignen (vgl. Abbildung 3). In der Informationswirtschaft und dem Verarbeitenden Gewerbe trifft dies auf etwa 73 % bzw. 66 % der Unternehmen zu, denen Crowdworking bekannt ist. Die fehlende Eignung der Arbeitsinhalte war bereits in den vorherigen Befragungswellen der am häufigsten genannte Hemmnisfaktor. Die fortwährenden Erweiterungen des Angebots und der Funktionalität von Crowdworking-Plattformen scheinen die Wahrnehmung der Unternehmen demnach nur in geringem Maße geändert zu haben.

Ein weit verbreitetes Hemmnis sehen die Unternehmen auch in Schwierigkeiten, die sich bei der Qualitätskontrolle der über eine Plattform bezogenen Leistungen ergeben können (65 % in der Informationswirtschaft und 60 % im Verarbeitenden Gewerbe). Mehr als die Hälfte derer, denen Crowdworking ein Begriff ist, sehen zudem in der Unsicherheit über juristische Rahmenbedingungen ein Hindernis. Demnach scheinen etwa arbeitsrechtliche Unsicherheiten nicht nur häufig auf der Angebots-, sondern auch auf der Nachfrageseite zu bestehen. Knapp 54 % der Unternehmen in der Informationswirtschaft und sogar 72 % im Verarbeitenden Gewerbe befürchten schließlich, dass die Vergabe von Aufträgen an die Crowd zu einem Abfluss von unternehmensinternem Wissen führen könnte.

In den vergangenen fünf Jahren haben sich die Hemmnisse der Nutzung von Crowdworking-Plattformen trotz mancher Schwankungen nicht stark verändert. Eine Ausnahme stellt der Anteil an Unternehmen dar, denen Crowdworking bekannt ist, die eine Nutzung von Crowdworking-Plattformen aber grundsätzlich ausschließen. Sowohl in der Informationswirtschaft als auch im Verarbeitenden Gewerbe gaben 2020 rund 24 % der Unternehmen an, dass Crowdworking eine Arbeitsform ist, die sie nicht unterstützen wollen. In den Befragungswellen der Jahre 2016 und 2018 lag dieser Anteil noch bei jeweils über 40 %. Der Anteil an Unternehmen, die Crowdworking kategorisch ablehnen hat sich demnach zwischen 2018 und 2020 nahezu halbiert.

Dr. Daniel Erdsiek ist Senior Researcher am ZEW — Leibniz-Zentrum für Europäische Wirtschaftsforschung in Mannheim.


Literatur

Belletti, C., D. Erdsiek, U. Laitenberger und P. Tubaro (2021), Crowdworking in France and Germany, ZEW Expert Brief, 21–09.
Berg, J., M. Furrer, E. Harmon, U. Rani und M. S. Silberman (2018), Digital Labour Platforms and the Future of Work: Towards Decent Work in the Online World, ILO.
Bonin, H. und U. Rinne (2017), Omnibusbefragung zur Verbesserung neuer Beschäftigungsformen, Expertise im Auftrag des Bundesministeriums für Arbeit und Soziales, IZA Research Report, 80.
Bundesregierung (2021), Dritter Gleichstellungsbericht der Bundesregierung — Digitalisierung geschlechtergerecht gestalten, BT-Drucksache 19/30750.
Erdsiek, D., J. Ohnemus und S. Viete (2019), Crowdworking in Deutschland 2018: Ergebnisse einer ZEW-Unternehmensbefragung, Expertise im Auftrag des Bundesministeriums für Arbeit und Soziales.
Kässi O Lehdonvirta V Stephany F How Many Online Workers are there in the World? A Data-Driven Assessment, Open Research Europe 2021 1 53 10.12688/openreseurope.13639.4 PMC10445891 37645214
Maier, M. F., S. Viete und M. Ody (2017), Plattformbasierte Erwerbsarbeit: Stand der empirischen Forschung, BMAS Forschungsbericht, 498.
Ohnemus, J., D. Erdsiek und S. Viete (2016), Nutzung von Crowdworking durch Unternehmen: Ergebnisse einer ZEW-Unternehmensbefragung, BMAS Forschungsbericht, 473.
Pesole, A., M. C. Urzí Brancati, E. Fernández-Macías, F. Biagi und I. González Vázquez (2018), Platform Workers in Europe — Evidence from the COLLEEM Survey, Publications Office of the European Union.
Stephany F Dunn M Sawyer S Lehdonvirta V Distancing Bonus or Downscaling Loss? The Changing Livelihood of US Online Workers in Times of COVID-19 Journal of Economic and Human Geography 2020 111 3 561 573 10.1111/tesg.12455 PMC7361450 32834151
